# Siblings’ life aspirations in the context of Duchenne muscular dystrophy: a mixed-methods case-control study

**DOI:** 10.1186/s41687-022-00501-7

**Published:** 2022-09-10

**Authors:** Carolyn E. Schwartz, Elijah Biletch, Richard B. B. Stuart, Bruce D. Rapkin

**Affiliations:** 1 DeltaQuest Foundation, Inc., 31 Mitchell Road, Concord, MA 01742 USA; 2 Department of Medicine and Orthopaedic Surgery, Tufts University Medical School, Boston, MA USA; 3 Department of Epidemiology and Population Health, Albert Einstein College of Medicine, Bronx, NY USA

**Keywords:** Duchenne muscular dystrophy, Aspirations, Goals, Siblings, Case-control, Mixed methods

## Abstract

**Background:**

The inevitable and progressive loss of independence in Duchenne Muscular Dystrophy (DMD) patients may have an impact on their siblings’ life aspirations. The present cross-sectional case-control study investigated how aspirations differed between brothers and sisters of people with DMD and a stratified comparison group of nationally representative children/adults.

**Methods:**

A web-based survey was administered October through December 2020. Aspirations were measured using qualitative (open-ended) and quantitative (closed-ended) questions. Qualitative prompts asked participants about wishes, goals, and how they define quality of life (QOL) and were coded by six trained raters. Quantitative questions included 29 closed-ended goal-delineation items from the QOL Appraisal Profile_v2_. These data were analyzed using multivariate models adjusting for propensity scores (demographic differences) and testing for the effects of role (sibling or comparison), age, and role-by-age interactions.

**Results:**

The study sample of DMD sibling (n = 349) and comparison (n = 619) participants provided open-ended data on 968 wishes statements, 390 QOL-definition statements, and 328 goals statements. Inter-rater reliability (kappa = 0.77) reflected good agreement between raters. Results of both open-ended and closed-ended data, and of both unadjusted and adjusted analyses suggested that DMD siblings, with age, were more focused on DMD-related, family/community, intimacy, and work concerns than their peers. They were less focused on improving mood, independence, pragmatics, or subtle fine-tuning of problem-solving in life. In contrast, the comparison group was more focused on goals related to growth, purpose, and reflection. Some group differences were amplified amongst older siblings.

**Conclusion:**

This is the first study to evaluate DMD siblings’ aspirations in comparison to their peers. While there were many similarities between groups, the differences in aspirations between DMD siblings and their peers encompassed not just DMD, family/community, and intimacy, but also more work concerns. Directions for future quantitative and qualitative research are discussed.

**Supplementary Information:**

The online version contains supplementary material available at 10.1186/s41687-022-00501-7.

## Introduction

Duchenne Muscular Dystrophy (DMD) is a progressive and irreversible neuromuscular genetic disorder which occurs nearly always in males and is rare (i.e., 1 in 5050 live births [1–5]). Females can be carriers and may have mild symptoms, such as mild but progressive muscular weakness and increased serum creatin kinase levels [6]. The DMD phenotype in female carriers of a dystrophin mutation has a direct correlation with a skewed X-chromosome inactivation pattern [6]. Usually diagnosed by age 5, the disorder presents as delayed development across multiple domains [7]. Generally by middle childhood (i.e., age 10–12), people with DMD experience progressive muscle weakness, loss of ambulation [8], upper-limb function problems, and painful concomitant conditions such as scoliosis and muscular contractures [1]. On average by age 15, these children experience increased breathing difficulties and life-threatening heart and lung conditions [9]. DMD patients typically die in their late 30 s to early 40 s [9], although medical advances have increased life expectancies somewhat [10].

This inevitable and progressive loss of independence in DMD patients is hypothesized to have an impact on their brothers’ and sisters’ life aspirations. Although DMD siblings may not suffer from the same progressive disability, they, too, are impacted by the disease. As discussed in her poignant essay, Verberkt notes that the fragility of her brother and the pervasive sense of worry in the family led to different concerns, a sense of responsibility, an experience of troublesome emotions, and a different career path than she might have had if not confronted by DMD [11].

Other than such anecdotal reports, research on the impact of DMD on siblings is limited. Read and colleagues utilized qualitative interviews [12] and quantitative measures [13] from DMD siblings to characterize the impact, concluding that DMD has the potential to increase emotional problems in siblings but may also promote positive family adjustment, such as increased family cohesion, enhanced sibling personal maturity, and use of coping mechanisms [12]. Magliano and colleagues studied parent caregivers of DMD, and reported that about one-third of parents believed that DMD had a negative influence on the psychological well-being and social life of the unaffected siblings [14]. In earlier work done by our group, DMD parental caregivers reported that many siblings gave up time with friends, sports or extracurricular activities, and/or summer camp or travel [15]. The caregivers also reported that there were insufficient finances for siblings’ activities or schooling, but tended to disagree that, due to DMD in the family, siblings had lost other opportunities, chosen only colleges close to home, or given up on going to college [15].

To our knowledge, no research has been done on life aspirations of siblings of people with DMD, and whether or how they differ from the general population. Going beyond what has traditionally been done in research on the family impact of caregiving, the present study investigates this impact by expanding the scope of the idea of burden. Specifically, we address a comprehensive idea of aspirations in DMD siblings as compared to those of a stratified comparison group of nationally representative children, teens, and adults. Our conceptualization triangulates on the comprehensive idea of aspirations by including emotional yearnings regardless of whether they are attainable (wishes [16]), personal aims (goals [17]), and what the individual perceives as central to a good quality of life [18] (QOL)(QOL definition). Qualitative and quantitative data were collected to address the research questions.

## Methods

Our companion paper describes fully the methods used for this web-based study [19]. We briefly describe the same for the sake of completeness of the present work [19].

### Sample and procedure

This study recruited DMD siblings via their parents who participated in an earlier study of DMD caregivers (see [15] for details). Comparison-group participants were recruited via IPSOS-Insight and were selected to accurately represent the United States in distributions of age, race/ethnicity, gender, and region. Eligible participants were children aged 8 and older to adults (aged 18 and over) and able to complete an online questionnaire. The protocol was reviewed and approved by the New England Independent Review Board (NEIRB #20,201,623), and all participants provided informed assent (if younger than 18) and/or consent (for parents of minor children or adult siblings) before beginning the survey.

### Measures

*Accommodating Age and Disability* The study design tailored the measures collected by age and/or participant preference. DMD siblings were offered the option to choose a simpler form of the survey if they felt they had trouble reading or concentrating. This “Alternate” survey was the same as the Child survey and asked fewer questions. Additional file 1: Table 1 shows the questions administered by survey type.

*Aspirations* were measured using qualitative (open-ended) and quantitative (closed-ended) questions to triangulate on the concept of aspirations. The open-ended questions included: (1) Three Wishes [20], in which participants were asked, “If you could make three wishes, any three wishes in the whole world, what would they be?”; (2) Goals: “What are the main things you want to accomplish?”; (3) QOL Definition: “In a sentence, what does the phrase ‘Quality of Life’ mean to you at this time?” The latter two are part of the QOL Appraisal Profile_v1_ [21].

In addition to the open-ended questions, 29 closed-ended goal-delineation items from the QOL Appraisal Profile_v2_ [22] (QOLAP_v2_) were included. These rating-scale items queried a broad range of life domains (e.g., living situation, work/school, social relationships, health-related, spiritual, etc.), asking the respondent how much each goal statement was like them (1 = “not at all like me” through 5 = “very much like me”). Participants were given the option of not responding (Not applicable/Decline/I don’t know), which was coded as missing (− 99). [The interested reader can contact the corresponding author for the QOLAP_v2_ Goal-Delineation items.]

Participants of all ages answered the Three Wishes open-ended question; DMD sibling participants aged 18 and older who did not opt for the Alternate survey answered the open-ended goals and QOL-definition questions and the closed-ended goal-delineation items.

*Demographic Characteristics* asked of all participants included year of birth, gender, whether received help with survey, height, weight, race, ethnicity, with whom the person lived, and whether anyone in the household was or had been infected with the novel coronavirus-2019. Adult participants were additionally asked about education level, marital status, difficulty paying bills, employment status, number of hours worked per week, occupational complexity, and hours missed from work from the Work Productivity & Activity Impairment [23]. Referring caregiver was tracked via the web recruitment link.

### Statistical analysis

#### Coding open-ended data

The open-ended data, assembled into a data set that included responses from patients, siblings, and the comparison group, were coded into themes by six trained raters (EB, RBB, AD, JBL, EK, MCF) according to a standardized protocol and comprehensive codebook derived from an extensive sorting procedure [24]. Themes were coded as “1’’ or ‘‘0’’ depending on whether they were reflected in the individual’s written text. A set of 40 themes was used for both the wishes and goals prompts and 17 for the QOL definition prompt. For each prompt, a theme of ‘‘No Direct Answer’’ was used if the respondent did not provide an answer or answered a different question than the one asked. For example, in response to the question ‘‘What are the main things you want to accomplish?’’ exemplary No-Direct-Answer responses were ‘‘many things” and ‘I've asked myself that question since I was a kid, and even now I have no idea’’.

Each text entry could be coded for as many themes as were identified, among the set for the corresponding prompt. Thus, one entry could elicit one theme or more than one depending on how the individual worded it. For example, as a goal one individual wrote, ‘‘My bills paid, my family healthy and happy, and family go to church.’ It was coded as reflecting family welfare, financial concerns, health issues, mental health/mood state, and religious/spiritual concerns. In contrast, another individual’s goal was ‘‘Move to a different state,” which was coded only with the single theme of living situation. Thus, we are assuming that the relevant factor here is the coded themes, not the individual wishes, goals, or QOL definitions themselves.

Training took place in two multi-hour sessions to understand the protocol and to utilize fully the codebook where themes were described fully and exemplified. Raters coded an initial set of ten participants’ data (from all three prompts), followed by a discussion of difference decisions across raters. They then coded the next ten participants’ data (again all prompts), and comparison and discussion now revealed almost no differences across raters. Raters then coded data from 40 more responses (all prompts), from which inter-rater reliability was computed in two ways on the 240 test responses (6 raters * 40 participant entries).

#### Inter-rater reliability

Two methods were used to assess aspects of inter-rater reliability. The first, Fleiss’s kappa [25] computed based on the entire data set, assessed degree of agreement over and above what would be expected by chance. This variant on the more familiar Cohen’s kappa [26] is used in cases of more than two raters. While there are no generally accepted rules of thumb for a desirable level of either form of kappa, some healthcare researchers have proposed values from 0.41 to 0.60 as “moderate,” 0.61–0.80 as “good,” and 0.81–1.00 as “very good.”[27, 28]

The second method assessed what proportion of the variance could be explained by the Rater effect. A low number is preferable as it reflects that the scores relate to the individual’s data being coded rather than reflecting a response style of the rater. This method used logistic regression to assess level of agreement among raters, with each of 240 “0” or “1” values regressed on the Rater variable, with its six categories (i.e., six raters). High inter-rater reliability (IRR) for any given theme would be indicated by a nonsignificant Rater effect, and one that explained a low fraction of the variance in ratings (as estimated by a pseudo-R-squared in the low single digits).

#### Comparing length, number of themes, and inter-method associations

Analysis of Variance (ANOVA) models were used to compare length of open-ended response and number of themes (dependent variables) by role (sibling vs. comparison; independent variable). Longer open-ended responses would generally reflect more complex or comprehensive answers.

#### Propensity scores

Demographic differences between DMD siblings and comparison participants were controlled in eventual multivariate models using propensity scores [29]. The goal of the propensity-score modeling was to create a score for covariate adjustment across all age groups, thereby allowing us to compare aspirations across the age span. This was the central contribution of the present work. We thus used the following pragmatic approach for dealing with the fact that some covariates were simply not asked and thus not available (see Additional file 1: Table 1). Accordingly, our propensity-score model adjusted for those covariates that differed between sibling and comparison groups in bivariate analyses described below. Separately for adults and for teens/children, a logistic regression model was computed predicting the dependent variable role (DMD sibling or comparison) from applicable covariates. For adults who completed the adult survey, the covariates included the following: ethnicity, White race, Black race, region, marital status, difficulty paying bills, whether currently working, education, whether received help completing survey, and whether someone in household had contracted COVID-19. For children, teens, and those who completed the Alternate survey, the covariates included the following: ethnicity, White race, Black race, region, whether received help completing survey, and whether someone in household had contracted COVID-19. For a small proportion of participants (6%), propensity scores were based on the mean propensity score among the individual’s age group).

#### Differences in age distributions by role

The child, teen, and adult data sets revealed age differences between patients and comparisons and between siblings and comparisons: there were differences in mean age, the frequency of certain age ranges, and the shape of the age distributions. We decided that, in addition to adjusting for age, in our models we would apply weights so as to simulate more comparable age distributions. While the weighting might not completely eliminate the age differences, it was aimed at making those distributions comparable enough to render the planned analyses tractable [30].

#### Analyzing the aspirations data

Descriptive statistics summarized either the proportion of each group coded as reflecting a given theme, for the open-ended data, or the central tendency, for the closed-ended goal-delineation items. Effect size was summarized by phi for comparison of proportions or Cohen’s *d* for comparison of means, the open- and closed-ended data, respectively. Multivariate models were then computed to hone the contrasts, comparing siblings vs. comparison group. In models, weighted to nearly equalize age, the two groups were contrasted in terms of role, age, and the role-by-age interaction, after adjusting for their propensity scores. Logistic regression was used to analyze the individual themes for the coded open-ended data, while Analysis of Covariance (ANCOVA) was used to analyze the closed-ended goal-delineation data.

For logistic regressions, 15 of the 95 theme variables showed no variation and thus were excluded from analyses. Further, some themes analyzed showed complete or quasi-complete separation in logistic regression, and for these we reported only descriptive results.

#### Interpretation of main effects in the context of interactions

The abovementioned multivariate models aimed to investigate how siblings compared to comparison participants in their aspirations and at different ages, after adjusting for demographic variables that might have confounded the raw descriptive comparisons. Interpreting main effects can be challenging when the model contains interaction terms, because the latter are collinear with the former. To address this challenge, plots of substantial interaction effects were used to facilitate their interpretation. In order to display an interaction effect (role*age), we created scatterplots that graphed predicted values from the entire model (Y-axis) against age (X axis), with separate lines for each Role group. Any theme variable with a group mean that was < 0.01, or with a |Beta| or |Estimated Beta| out of the usual range (> 1.3), was excluded from interaction graphs.

#### Interpretation in the context of many contrasts

The present study involves a large number of statistical contrasts, primarily because it is investigating research questions that have not been addressed to date and which involve translating nuanced qualitative data into quantitative metrics. Beyond demographic comparisons, where we rely on p-values to identify group differences, we focused our interpretation on effect sizes (ES). Cohen’s criteria were used to facilitate interpretation of differences for medium and large effect sizes, respectively: in proportions (Phi of 0.3 and 0.5); in mean differences (Cohen’s *d* of 0.5 and 0.8); in model explained variance (R^2^ of 0.06 and 0.14); and in model parameter estimates (standardized coefficients or β of 0.3 and 0.5). While we report ES regardless of magnitude, we interpret only medium or large ES because these are generally considered clinically important [31]. Tables are conditionally formatted using data bars in unadjusted comparisons, and using different colors and saturation levels in adjusted comparisons, to highlight effects’ direction and magnitude.

IBM SPSS version 27 [32] and the R software [33] were used for all analyses.

## Results

### Sample

The study sample included 349 siblings of people with DMD and 619 comparison participants. Table 1 shows descriptive information about the two subsamples, as well as results of analyses contrasting the groups on all demographic variables. The majority of the sample was Caucasian, 6–20% per Role group were Black, and 8–20% were Hispanic. Both subsamples had a normal body mass index on average. Study participants in both groups were young adults from a variety of regions of the contiguous United States.

**Table 1:** Sample Demographic Characteristics (N = 968)

		Sibling (N < = 349)		Comparison (N < = 619) ¥		Cohen's *d*	Cramer's V	*p*-value
Variable		Mean	SD	Mean	SD			
Age		18.2	5.8	19.1	7.6	− 0.14		0.06
Body mass index		24.2	5.4	23.6	6.0	0.10		0.20
Variable		#	%	#	%			
Gender**							0.02	0.85
	Male	168	48%	292	47%			
	Female	177	51%	324	52%			
	Other	1	0%	1	0%			
	Missing	0		0				
Living alone*	Yes	2	1%	4	1%		0.01	0.91
Marital status (if age ≥ 18)							0.35	< 0.0005
	Never married (or no response)	135	93%	136	62%			
	Married	7	5%	50	23%			
	Cohabitation	1	1%	26	12%			
	Separated	1	1%	4	2%			
	Divorced	1	1%	3	1%			
	Widowed	0	0%	0	0%			
Ethnicity*	Hispanic or Latino	28	8%	121	20%		0.15	< 0.0005
Race (check all that apply)**								
	White	314	90%	456	74%		0.19	< 0.0005
	Black or African American	21	6%	123	20%		0.19	< 0.0005
	Other	6	2%	72	12%		0.18	< 0.0005
	Missing	10		4				
United States Region							0.15	0.01
	East North Central	37	11%	93	15%			
	East South Central	36	10%	46	7%			
	Middle Atlantic	26	7%	77	12%			
	Mountain	25	7%	38	6%			
	New England	12	3%	23	4%			
	Pacific	65	19%	78	13%			
	South Atlantic	86	25%	127	21%			
	West North Central	16	5%	29	5%			
	West South Central	29	8%	71	11%			
	Non-Contiguous	0	0%	0	0%			
	missing	17	5%	37	6%			
Difficulty paying bills (if age > 18)**							0.34	< 0.0005
	Not at all difficult	103	77%	94	45%			
	Slightly difficult	17	13%	40	19%			
	Moderately difficult	8	6%	48	23%			
	Very difficult	2	2%	20	9%			
	Extremely difficult	3	2%	9	4%			
	Not applicable / missing	216		408				
Employment status (if age > 18)**							0.25	< 0.0005
	Employed	57	42%	130	61%			
	Unemployed	76	56%	71	33%			
	Retired	0	0%	8	4%			
	Disabled due to medical condition	2	1%	3	1%			
	Missing	214		407				
Education (if age > 18)**							0.37	< 0.0005
	Less than 12th grade	4	3%	6	3%			
	High school diploma	19	13%	60	27%			
	Some college	58	40%	50	23%			
	Technical (Vocational) degree	36	25%	17	8%			
	4-year University degree	24	17%	62	28%			
	Masters degree	2	1%	20	9%			
	Doctoral or professional degree	1	1%	5	2%			
	Missing	205		399				
Had help completing survey*	Yes	90	26%	119	19%		0.08	0.02
Participant or a household member had COVID-19**							0.26	< 0.0005
	Definitely or probably Yes	4	1%	113	19%			
	No	339	99%	495	81%			
	Missing	6		11				

Some sets of percentages may not add up to 100% due to rounding

*GED* General Educational Development (i.e., high-school equivalency test), *SD*  Standard deviation

^*^For these variables a non-response was counted as the absence of the characteristic in question

^**^For these variables Cramer's V and p are based on non-missing results

¥ Comparison participants included only if age < 35

DMD siblings were 48% male and had a median level of education of some college. Fifty-six percent of DMD adults were unemployed, 1% were unable to work due to their medical condition, and 42% were employed. About 1/4 received help completing the survey (Table 1). Adults in this group endorsed no difficulty or slight difficulty paying bills (90%), almost all were unmarried, and 1% lived alone. Only 1% reported that a household member had contracted COVID-19.

The comparison subsample also had a median level of education of some college, but a higher proportion reported completing a 4-year college degree or graduate/professional degree (Table 1). Sixty-one percent were employed, 33% unemployed, and 1% were unable to work due to a medical condition. Sixty-four percent of comparison adults endorsed little or no difficulty paying bills. Sixty-two percent reported being unmarried, and only 1% lived alone. Nineteen percent reported that a household member had contracted COVID-19. The same fraction received help completing the survey.

Analyses contrasting these two groups on the demographic variables revealed significant differences (*p* < 0.05) on marital status, ethnicity, race, United States region, difficulty paying bills, employment status, education, whether a household member had contracted COVID-19, and whether they received help with the survey (Table 1). The siblings reported less financial strain and a substantially lower incidence of COVID-19 in the household (Table 1). Siblings were more likely to receive help completing the survey.

### Qualitative coding reliability

The open-ended data included 968 wishes statements, 390 QOL-definition statements, and 328 goals statements. Inter-rater reliability was computed for a randomly selected 17 topics across these three prompts. The mean kappa across the topics was 0.77 (SD = 0.17, range 0.51–0.98), reflecting a good level of agreement [27, 28]. The best estimates for pseudo-R^2^ for rater, averaged over the Cox & Snell and Nagelkerke methods, had a mean of 0.021 (range 0.001–0.104), with 0.17 < *p* < 1.00, suggesting that any rater effect on the coded themes was negligible.

### Propensity scores

Additional file 1: Table 2 shows model descriptive statistics as well as parameter estimates for all covariates, from the propensity-score model distinguishing siblings from comparisons. Our propensity-score model adjusted for those covariates that differed between sibling and comparison groups (see Table 1). Averaged across iterations, the model explained 31% of the variance as estimated via pseudo-R^2^.

### Aspiration differences by role: univariate results

#### Open-ended data

Table 2 provides unadjusted group differences on the themes coded in the three open-ended aspirations prompts. Only one theme showed a medium effect-size difference in proportion by role (|Phi|≥ 0.3). Unadjusted estimates show that siblings were much more likely than comparison participants to mention wishes related to DMD.

**Table 2:**
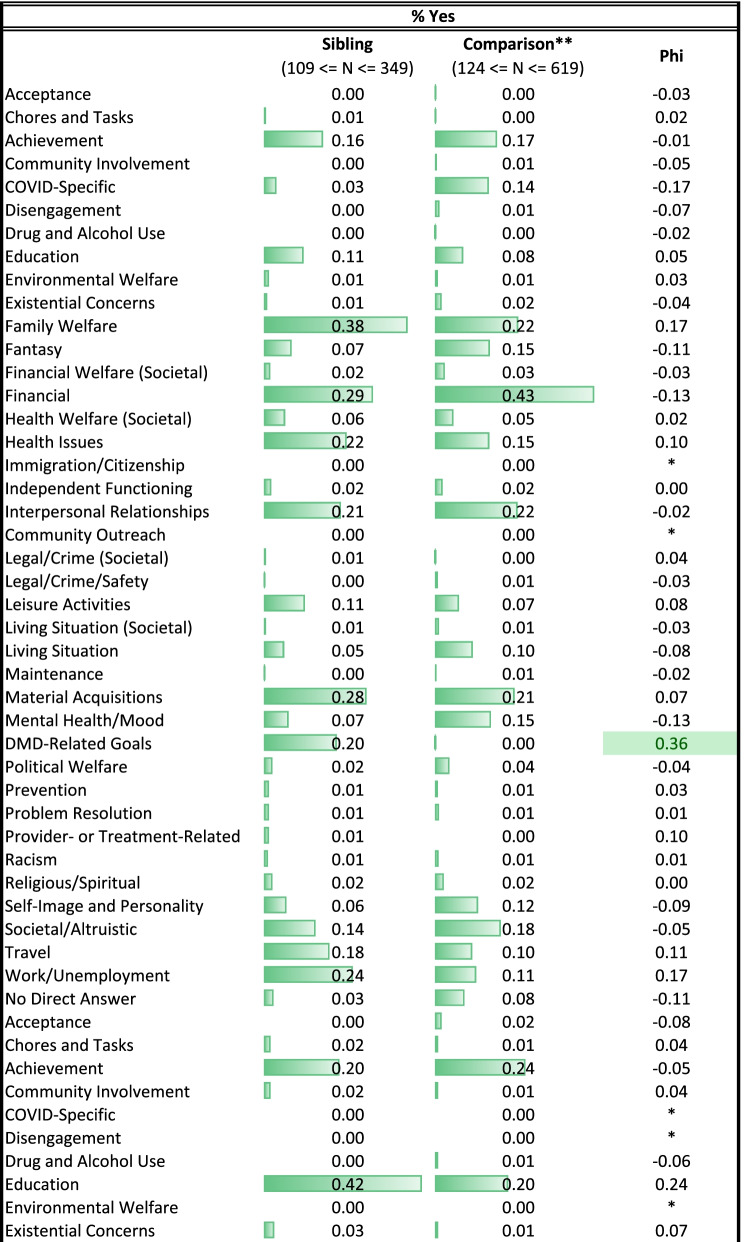

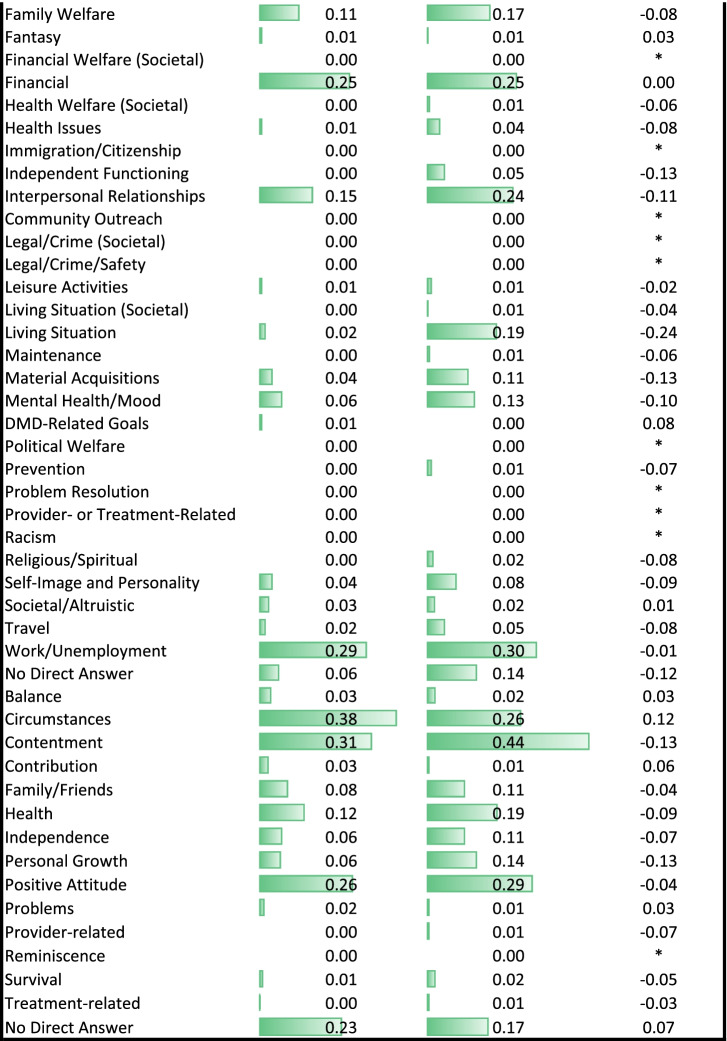
Descriptive statistics for themes from open-text prompts

#### Closed-ended goal items

**Fig. 1:**
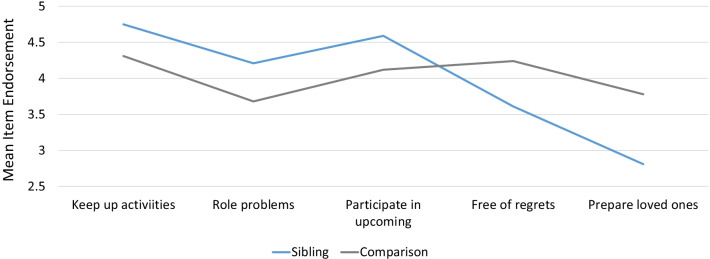
Goal Item Endorsement by Role**.** Unadjusted means are shown for siblings (blue line) and comparison participants (grey line) on the closed-ended goal items with the most substantial differences

Table 3 provides unadjusted group differences for the closed-ended goal items by role. Figure 1 shows unadjusted mean differences on the items that showed the most substantial differences (|Cohen’s d|≥ 0.50) between groups. Siblings were more concerned with keeping up activities, solving problems related to carrying out roles that are important to them, such as at work, school, homemaking, or volunteering, and participating in important upcoming events; and less concerned with being free of regrets and preparing their loved ones for a time when their health would be worse.

**Table 3:**
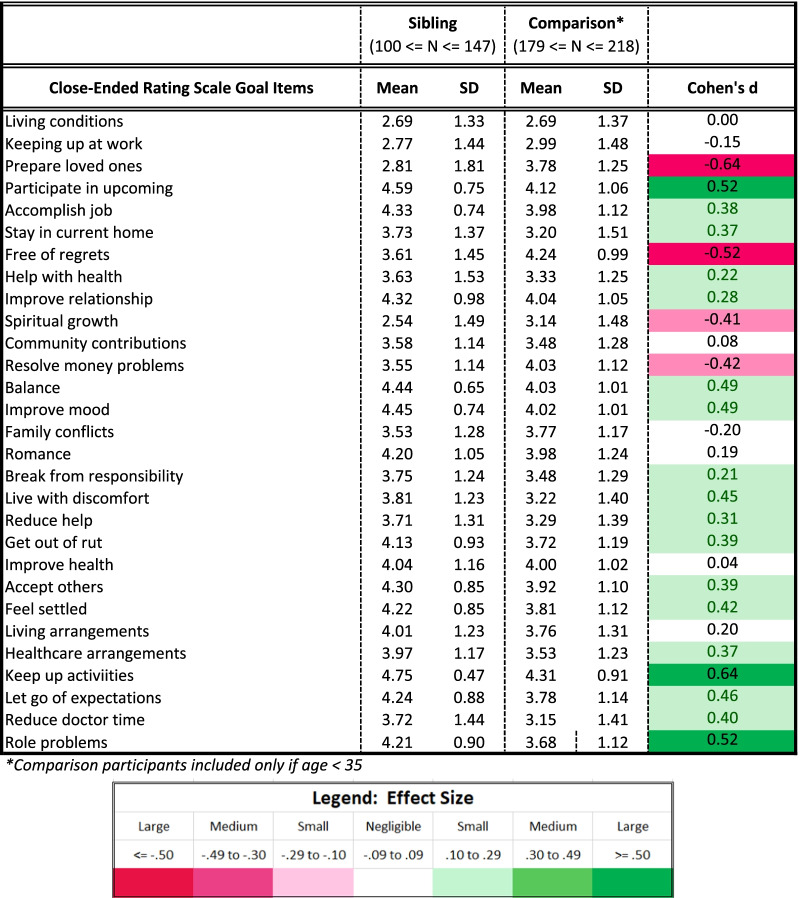
Descriptive statistics for close-ended goal items

### Aspiration differences by role: multivariate results

#### Open-ended data

**Fig. 2:**
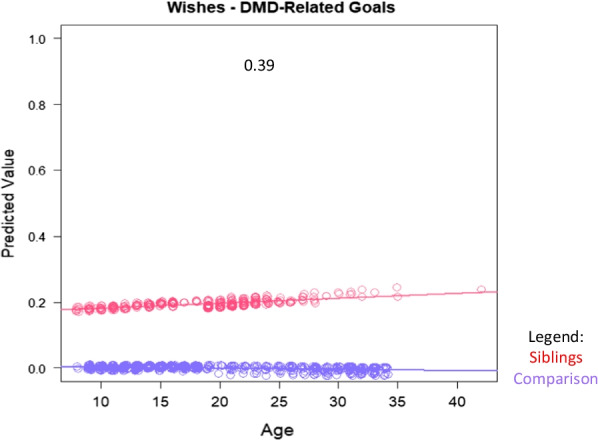
Interaction plots for adjusted logistic models predicting wishes themes. Role-group differences for medium- and large-ES interaction effects are shown. Sibling predicted values are shown in red; comparison in blue

**Fig. 3:**
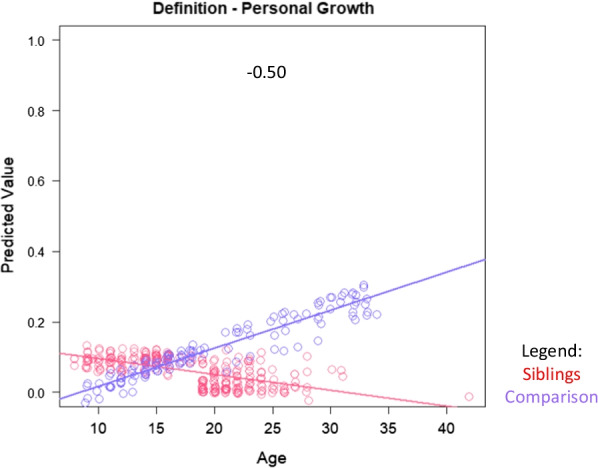
Interaction plots for adjusted logistic models predicting QOL definition themes**.** Role-group differences for medium- and large-ES interaction effects are shown. Sibling predicted values are shown in red; comparison in blue

**Fig. 4:**
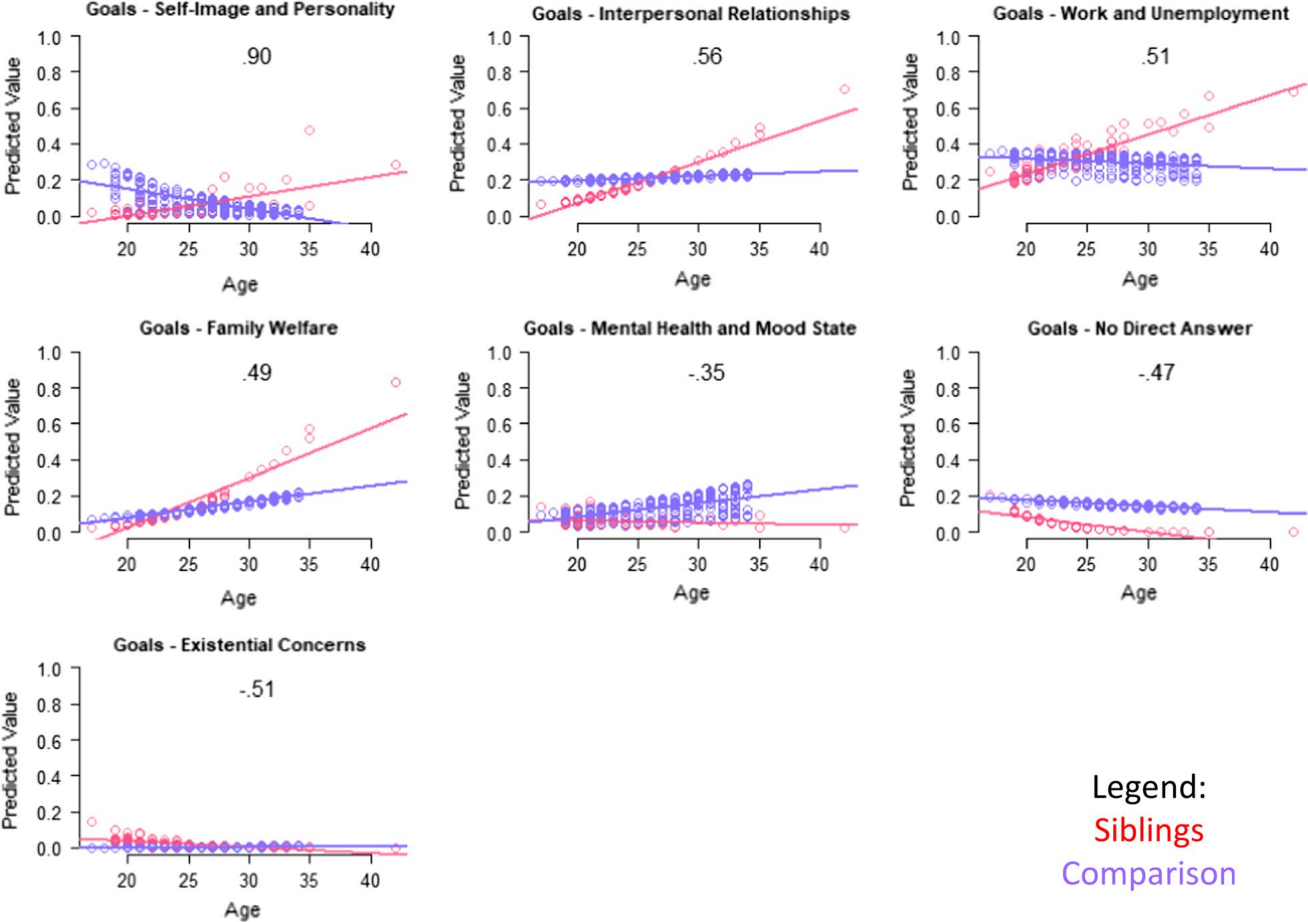
Interaction plots for adjusted logistic models predicting Goal themes. Role-group differences for medium- and large-ES interaction effects are shown. Sibling predicted values are shown in red; comparison in blue

Table 4 shows results of the multivariate models comparing siblings and comparison participants on coded themes from their open-ended Aspirations data, after adjusting for age, role-by-age interactions, and propensity scores. Again, siblings were more likely to express DMD-related wishes, particularly with age (Fig. 2). As a main effect, siblings were more likely than comparisons to define QOL in terms of personal growth, but this difference was reversed among adults (Fig. 3). Siblings were, as a main effect, less likely than comparisons to mention goals related to self-image and personality, but this difference, too, reversed itself among older participants (Table 4 and Fig. 4, respectively). Older siblings were more likely than comparison counterparts to bring up interpersonal relationships, work/unemployment, and family welfare (Fig. 4). In contrast, older comparison participants were the group more likely to write about mental health/mood state and existential concerns, or to give no direct answer (Fig. 4).

**Table 4:**
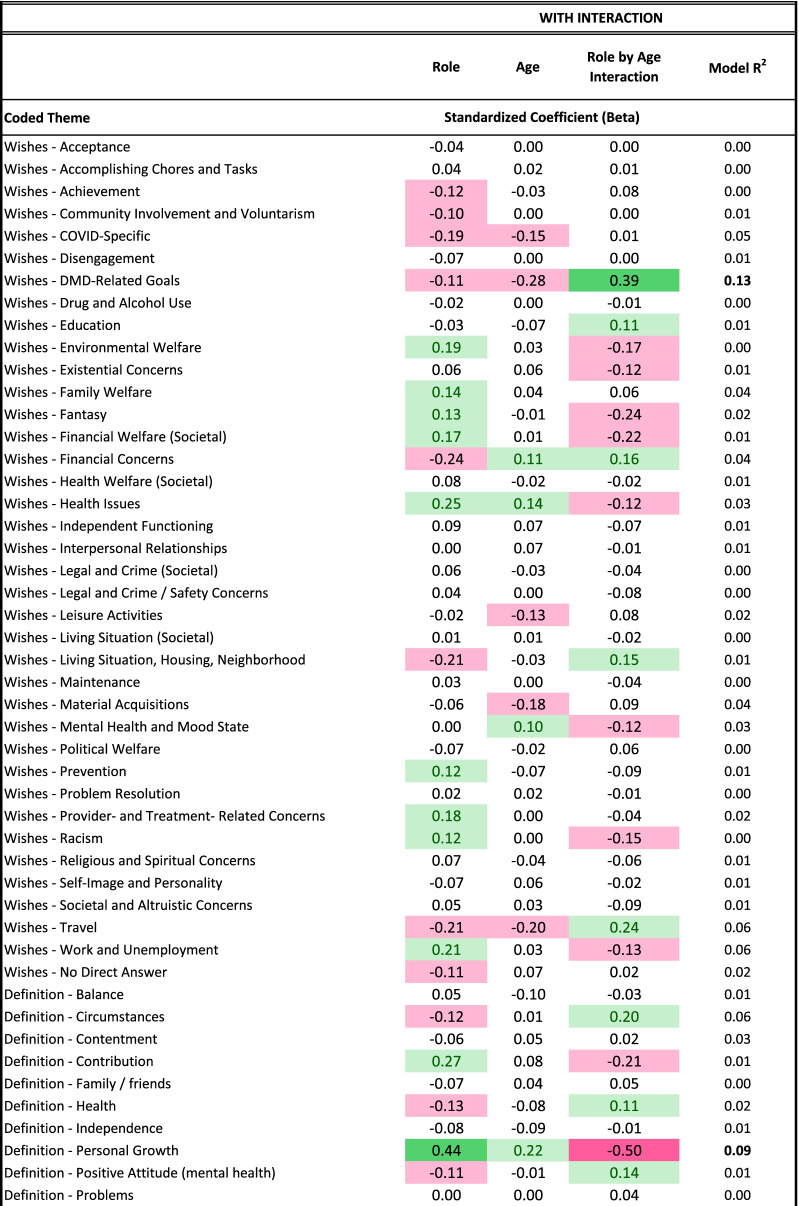

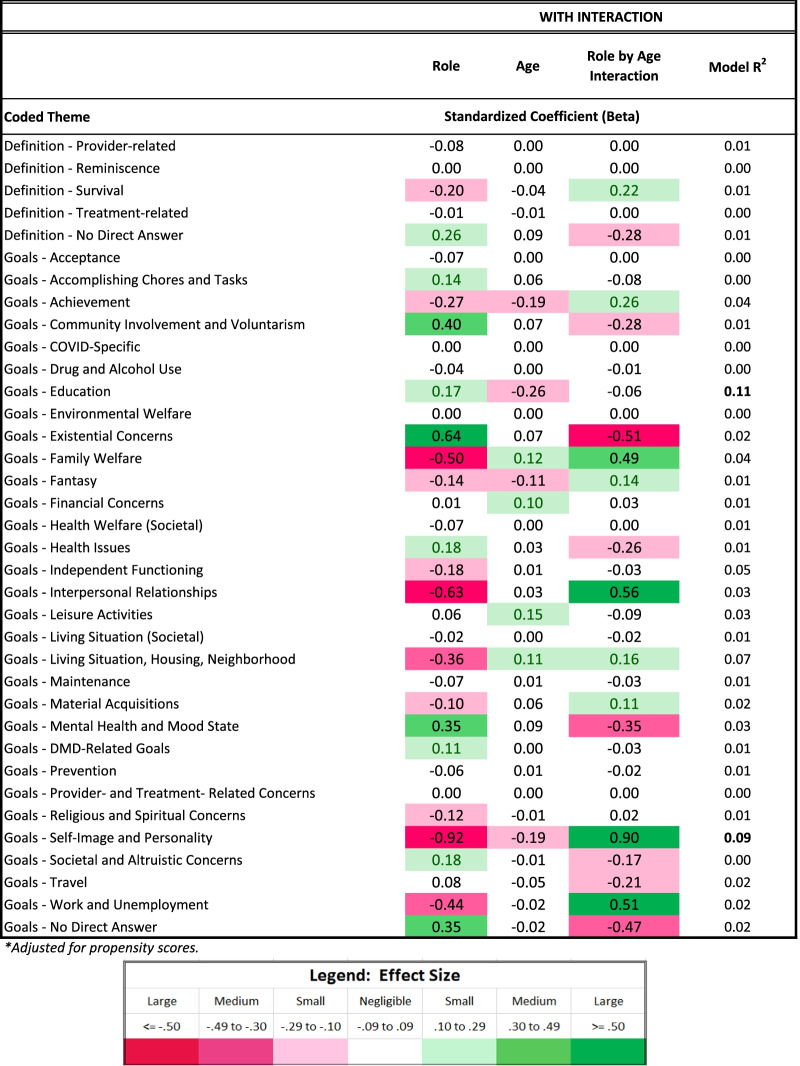
Results of sibling vs. comparison groups' multivariate logistic models predicting coded themes*

#### Closed-ended goal items

**Fig. 5:**
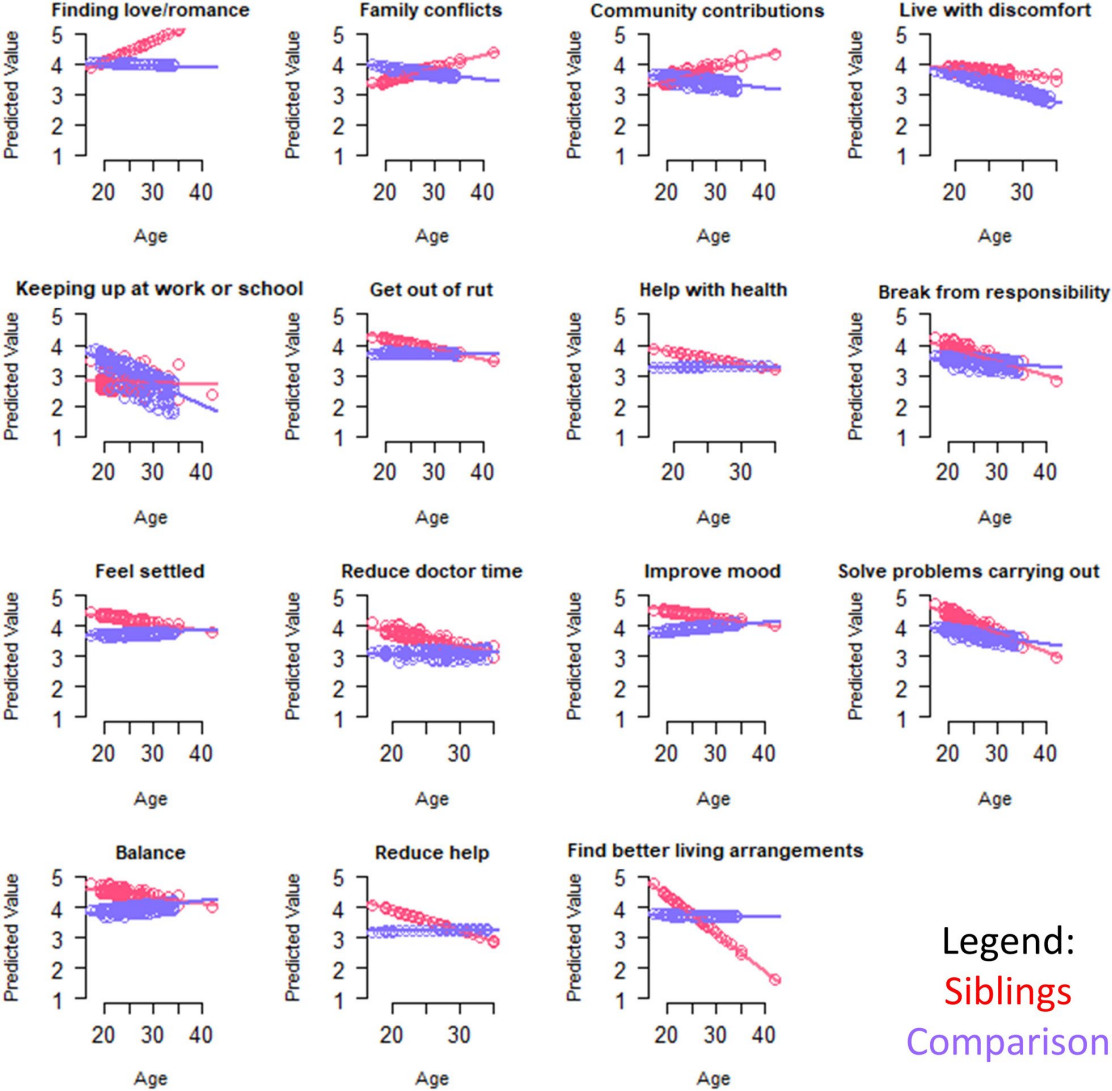
Interaction plots for adjusted ANCOVA models predicting Goal items. Role-group differences for medium- and large-ES interaction effects are shown. Sibling predicted values are shown in red; comparison in blue

Table 5 shows results of the multivariate models comparing groups on closed-ended goal items, after adjusting for propensity scores, age, and role-by-age interactions. Goals that became more important with age for siblings, as reflected by positive role-by-age interactions, were related to finding love/romance, resolving family conflicts, community contributions, and living with discomfort (Fig. 5). Goals that became less important with age for siblings were related most sharply to finding better living arrangements. Additionally, older siblings were less concerned with getting out of a rut, getting help with health, getting a break from responsibilities, feeling settled, reducing time spent with doctors, improving mood, solving problems carrying out roles, having balance, and reducing help from others (Fig. 5).

**Table 5:**
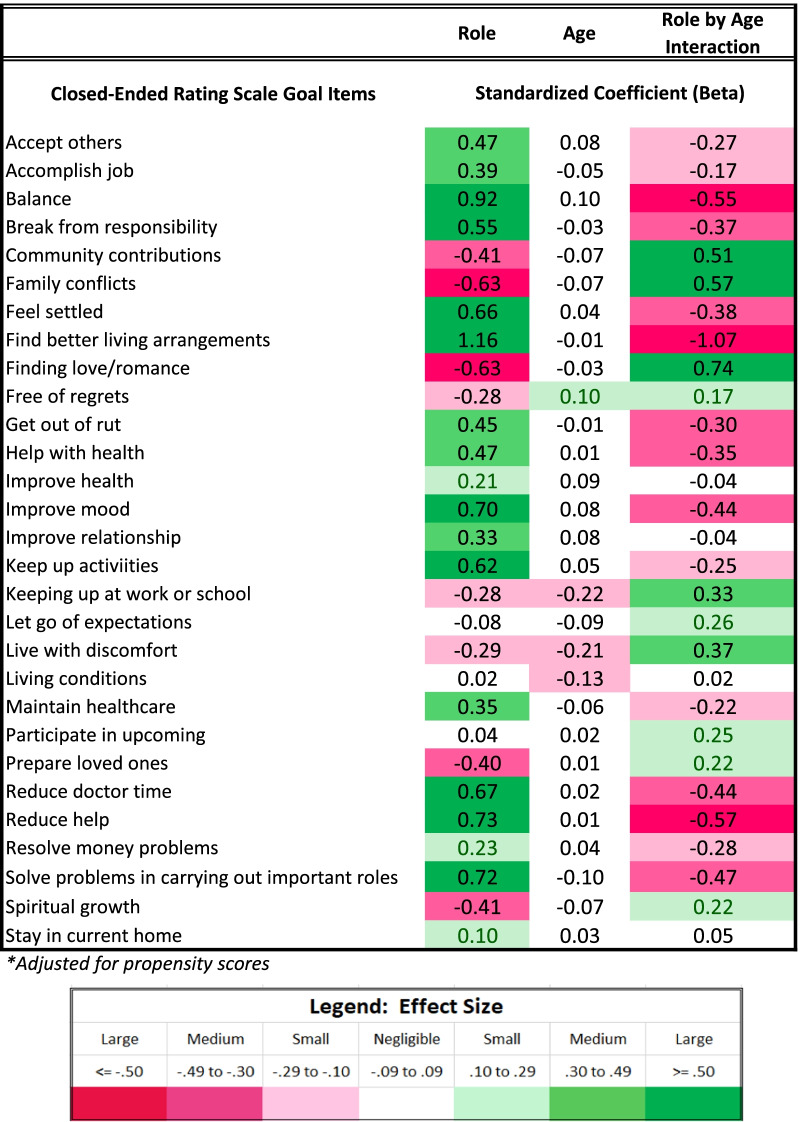
Results of sibling vs. comparison groups' ANCOVA models predicting closed-ended goal items*

## Discussion

To our knowledge, this is the first study to address aspirations in siblings of people with DMD. In comparison to a general-population sample of similar age peers, we found that DMD siblings were more likely to focus on DMD-related wishes and goals related to family/community, intimacy, work, living with discomfort, and self-image. They were less focused on improving mood, independence, pragmatics, or subtle fine-tuning of problems in life. The comparison group, on the other hand, was more focused on goals related to growth, purpose, and reflection.

It was interesting to note that compared to the patient results reported in our companion paper [19], there were substantially fewer differences between siblings and the comparison participants,^1^ and these differences revolved almost entirely around goals rather than other aspects of aspirations. There were important ways in which the experience of growing up in a family with DMD children appeared to affect the non-DMD children. Particularly with age, that experience is associated with a greater focus on family/community and interpersonal concerns rather than pragmatic or operational concerns or on matters relating to health or to receiving help. Thus, despite the burden associated with having a DMD sibling, the siblings in our study exhibit a notable resilience: they have many similar aspirations to their peers despite the DMD context. The siblings also exhibit altruism and other-centeredness in their aspirations, in comparison to peers. Their prominent focus on their brother’s health and family welfare suggest core intrinsic values, such as those centered on relationships, personal well-being, and community [34]. These findings show some similarity with research on hemophilia caregivers that suggested that adopting positive ways of thinking about one’s life limitations may transform the negatives of caregiving into something positive [35].

This study focused on description, and explicitly avoided interpreting the valence of such differences. Understanding whether revealed differences are negative or positive would require another set of analyses. Future research could, for example look at correlations among aspiration themes and QOL outcomes collected cross-sectionally or longitudinally. Building on the present work, future research might also implement in-depth interviews over time to understand the impact of having a sibling with DMD on key decision points in the well sibling’s life. For example, one could examine whether siblings accomplish their stated aspirations, whether their achievement is consistent with their educational background, and/or whether their achievements diverge from peers at a later age perhaps due to compromises in the face of family demands.

The present study has many advantages. In addition to including a comparison cohort of peers, it also triangulates on a comprehensive idea of aspirations across the lifespan from middle childhood to middle age, by including emotional yearnings, personal aims, and what the individual perceives as central to a good quality of life. Its large sample size enables rigorous analysis and consideration of developmental changes. The use of both qualitative and quantitative measurement methods enable an in-depth study of aspirations.

Nonetheless, the study’s limitations should be acknowledged. First, the cross-sectional, quasi-experimental design limits causal inference. Although having a comparison group enables some inference about differences, we cannot unequivocally attribute differences to the causal impact of DMD on the family, but rather their associated (i.e., correlated) differences. Second, because siblings were allowed to opt out of the more involved adult survey, information collected about adults is likely generalizable to more high-functioning siblings, and not to those with more cognitive challenges such as attention-deficit or developmental delays. Third, although multivariate comparisons were adjusted for propensity scores that considered demographic characteristics, there is some debate as to how well sizable pre-existing group differences can effectively be neutralized using multivariate control [30]. Further, the use of propensity scores in case–control studies may suffer from artifactual effect modification of the odds ratio by level of the propensity score and may not fully adjust for measured confounding factors [36]. Additionally, the missing-data imputation method used may underestimate the variance of the parameter estimates and thus inflate Type I errors [37]. Covariate adjustment using propensity scores assumes that the nature of the relationship between the propensity score and the outcome has been correctly modeled ([29], p. 409). The plots illustrating our findings show that the observed data points are close to the regression lines, thereby supporting the idea that the outcome has been correctly modeled and assuaging concerns about bias generated by the pragmatic approach used to generate propensity scores across the full age range of the study sample. If we had implemented models separately for those with and without adult-level covariates (e.g., marital status, difficulty paying bills, etc.), we would likely have missed important interaction effects. Although a mean imputation approach is not standard for propensity scores, there was only small amount of missing data. Thus, our pragmatic approach for handling this complex dataset structure enabled filling a gap in the literature. Finally, due to the number of variables generated from the content analysis of qualitative data, a large number of comparisons were done. We have attempted to mitigate the possible multiple-comparison issue by focusing on only medium and large ES using Cohen’s criteria [38]. Given the sample sizes of the two groups being compared, the study has sufficient statistical power to detect even a small ES with an alpha level of 0.05 [38]. Thus, medium and large ESs would have an even smaller Type I error rate. As this study presents for the first time to our knowledge novel results about how DMD siblings’ aspirations differ from a comparison sample, we believe that quantitatively considering all the data gleaned from the qualitative analysis is worthwhile and important.

### Conclusions

In summary, this first study addressing aspirations in siblings of people with DMD provides insight into important similarities and differences in aspirations between siblings of people with DMD and their peers. These differences encompass concerns related to DMD, family/community, intimacy, and work, and are less focused on pragmatic or operational concerns. Future research might build on this study by evaluating differences in achievement of such aspirations over time.

## Supplementary Information

**Additional file 1: Supplemental Table 1.** List of Measures by Age Cohort.

## Data Availability

The study data are confidential and thus not able to be shared.

## Abbreviations

ANCOVA: Analysis of covariance
ANOVA: Analysis of variance
DMD: Duchenne muscular dystrophy
ES: Effect size
IRR: Inter-rater reliability
QOL: Quality of life

## Footnotes

1 24 medium or large effect-size interactions for siblings versus 33 for patients.

## Code availability

Requests for software code will be considered.
