# Supplementary material for: Siblings’ life aspirations in the context of Duchenne muscular dystrophy: a mixed-methods case-control study
Source: J Patient Rep Outcomes. 2022 Sep 10;6:96. doi: 10.1186/s41687-022-00501-7 (PMC9463676; doi:10.1186/s41687-022-00501-7)
Supplement: Supplementary file 1 — Additional file 1: Supplemental Table 1. List of Measures by Age Cohort. [file 41687_2022_501_MOESM1_ESM.pdf]

Supplemental Table 1. List of Measures by Age Cohort					
**Sample Characteristics**		**Age Stratum**			
Study stratum		Younger	Older		*Alternate Survey*
Target developmental level for measure		Child	Teen	Adult	
Age range		8-12	13-17	>=18	
**Construct**	**Measure**				
**Aspirations**					
**Open-Ended**	Three Wishes	√	√	√	√
	QOLAPv1 Open-Ended Q's: QOL Definition (1)			√	
	QOLAPv1 Open-Ended Q's: Goals to accomplish (1)			√	
**Close-Ended**	QOLAPVv2 Long-Form: 29 goal items			√	
**Control Covariates**					
**Work Life**	Employment status, number of hours worked per week, occupational complexity, Work Productivity & Activity Impairment item			√	
**Demographics**	Year of birth, gender, received help with survey, height, weight, race, ethnicity, with whom the person lives, COVID infection in household	√	√	√	√
	education, marital status, difficulty paying bills			√	

** Consent for participants younger than 18 years of age was provided by parent, with assent from child.*

Confidential DeltaQuest Information. All rights reserved.

Supplemental Table 2. Propensity Score Models				
**Siblings vs. Comparisons (n=968)**				
**Model Descriptive Statistics**	Iteration 1	Iteration 2	Iteration3	**Mean**
Cox & Snell R Square	0.43	0.272	0.093	0.26
Nagelkerke R Square	0.58	0.364	0.133	0.36
**Parameter Estimates, Main Adult Model**	**B**	**S.E.**	**Sig.**	**Odds Ratio**
Marital status	1.14	0.29	<.0005	3.13
Ethnicity	2.18	0.65	0.001	8.85
Black	-2.41	1.80	0.18	0.09
White	-4.71	1.79	0.01	0.01
Region			0.24	1.00
East North Central	1.76	0.88	0.05	5.83
East South Central	-0.06	0.67	0.93	0.95
Middle Atlantic	0.67	0.79	0.40	1.94
Mountain	-0.31	0.72	0.67	0.73
New England	-0.47	0.72	0.51	0.62
Pacific	-0.64	0.95	0.50	0.53
South Atlantic	-0.74	0.72	0.31	0.48
West North Central	0.03	0.67	0.96	1.03
West South Central	-0.25	0.82	0.76	0.78
Difficulty paying bills	0.58	0.18	0.00	1.79
Currently working	-0.65	0.31	0.04	0.52
Education level	0.11	0.14	0.44	1.12
Received help completing survey	1.19	0.90	0.19	3.29
Definitely or probably had Covid	2.61	0.80	0.28	13.60
